# Measurement of nuclear reaction cross sections by using Cherenkov radiation toward high-precision proton therapy

**DOI:** 10.1038/s41598-018-20906-z

**Published:** 2018-02-07

**Authors:** Takamitsu Masuda, Jun Kataoka, Makoto Arimoto, Miho Takabe, Teiji Nishio, Keiichiro Matsushita, Tasuku Miyake, Seiichi Yamamoto, Taku Inaniwa, Toshiyuki Toshito

**Affiliations:** 1 Graduate School of Advanced Science and Engineering, Waseda University, Tokyo, Japan; 2 Department of Medical Physics, Tokyo Women’s Medical University, Tokyo, Japan; 3 Department of Radiology, Kyoto Prefectural University of medicine, Kyoto, Japan; 4 Graduate School of Science, Rikkyo University, Tokyo, Japan; 5 Graduate School of Medicine, Nagoya University, Nagoya, Japan; 6 National Institute of Radiological Sciences, QST, Department of Accelerator and Medical Physics, Chiba, Japan; 7 Nagoya Proton Therapy Center, Nagoya, Japan

## Abstract

Monitoring the *in vivo* dose distribution in proton therapy is desirable for the accurate irradiation of a tumor. Although positron emission tomography (PET) is widely used for confirmation, the obtained distribution of positron emitters produced by the protons does not trace the dose distribution due to the different physical processes. To estimate the accurate dose from the PET image, the cross sections of nuclear reactions that produce positron emitters are important yet far from being sufficient. In this study, we measured the cross sections of ^16^O(*p*,*x*)^15^O, ^16^O(*p,x*)^13^N, and ^16^O(*p*,*x*)^11^C with a wide-energy range (approximately 5–70 MeV) by observing the temporal evolution of the Cherenkov radiation emitted from positrons generated via *β*^+^ decay along the proton path. Furthermore, we implemented the new cross sectional data into a conventional Monte Carlo (MC) simulation, so that a direct comparison was possible with the PET measurement. We confirmed that our MC results showed good agreement with the experimental data, both in terms of the spatial distributions and temporal evolutions. Although this is the first attempt at using the Cherenkov radiation in the measurements of nuclear cross sections, the obtained results suggest the method is convenient and widely applicable for high precision proton therapy.

## Introduction

Proton therapy is one of several radiation therapies to treat cancer. The most important advantage of using protons is the depth of the dose distribution as the protons deposit the highest dose near the end of their paths, at the Bragg peak. Owing to these characteristics, the dose can be concentrated to a tumor while minimizing the damage to normal tissues. Moreover, the protons have a sharp dose gradient; however the accurate estimation of dose distributions in human bodies is difficult owing to the uncertainties in the proton range in the human body. In the treatment planning of proton therapy, the patients undergo an X-ray computed tomography (CT) of the irradiation area. The conversion of X-ray CT numbers into proton stopping powers has uncertainties of the order of a few percent, which is equal to a few millimeter uncertainties of the proton range^1–4^. In addition, the position, motion, and anatomical changes of a patient can produce even more uncertainties. For these reasons, normal tissues may be fatally damaged if appropriate irradiation is not performed or an insufficient dose is delivered to the tumor. It is thus preferable to monitor the actual dose distribution *in vivo*.

During proton therapy, an incident proton that exceeds the Coulomb barrier bombards a target nucleus in the human body. This process is called nuclear fragmentation. To detect the gamma rays from the annihilation, positron emission tomography (PET) is used to visualize the distribution of positron emitters during or after irradiation^5–16^. However, the physical processes that produce positron emitters via the nuclear reactions is different from the energy loss process of protons through the electromagnetic interaction; as a result, the PET image does not reflect the dose information along the proton path. To estimate the dose from the PET image, a spatial distribution of positron emitters corresponding to various proton dose distributions must be known a priori^17–22^. Therefore, cross sectional data of the nuclear reactions that produce positron emitters are important, particularly for the components of the human body, for e.g., oxygen, carbon, and calcium. However, the current database of such nuclear reactions is insufficient below 250 MeV, which are important for proton therapy^6–8,10,11,14,23,24^. The lower energy region (under ∼70 MeV) is especially important because decelerated protons damage tumors most effectively^14,23,24^. Moreover, as the proton flux rapidly decreases, the straggling effect becomes stronger, and the cross sections vary drastically. Because the nuclear cross sections reported by previous studies have large uncertainties with a limited energy range, the precise measurement for the wide energy range has not yet been performed.

In this study, we developed a novel method of measuring nuclear reaction cross sections toward high-precision proton therapy by using Cherenkov radiation after proton irradiation. We used an electron multiplying charge-coupled device (EM-CCD) camera as a detector to visualize weak Ceherenkov radiation from the irradiated target. A synthetic quartz glass SiO_2_, containing oxygen, was irradiated with 72.0-MeV proton. We acquired cross sections of ^16^O(*p,x*)^15^O, ^16^O(*p,x*)^13^N, and ^16^O(*p,x*)^11^C, and compared them with the archival data. In addition, we used the obtained cross sections with Monte Carlo (MC) simulation code, the Particle and Heavy Ion Transport Code System (PHITS). Finally, we present a quantitative comparison between the MC simulation and PET measurements of water to validate the proposed method.

## Results and Discussion

### Cherenkov image

**Figure 1:**
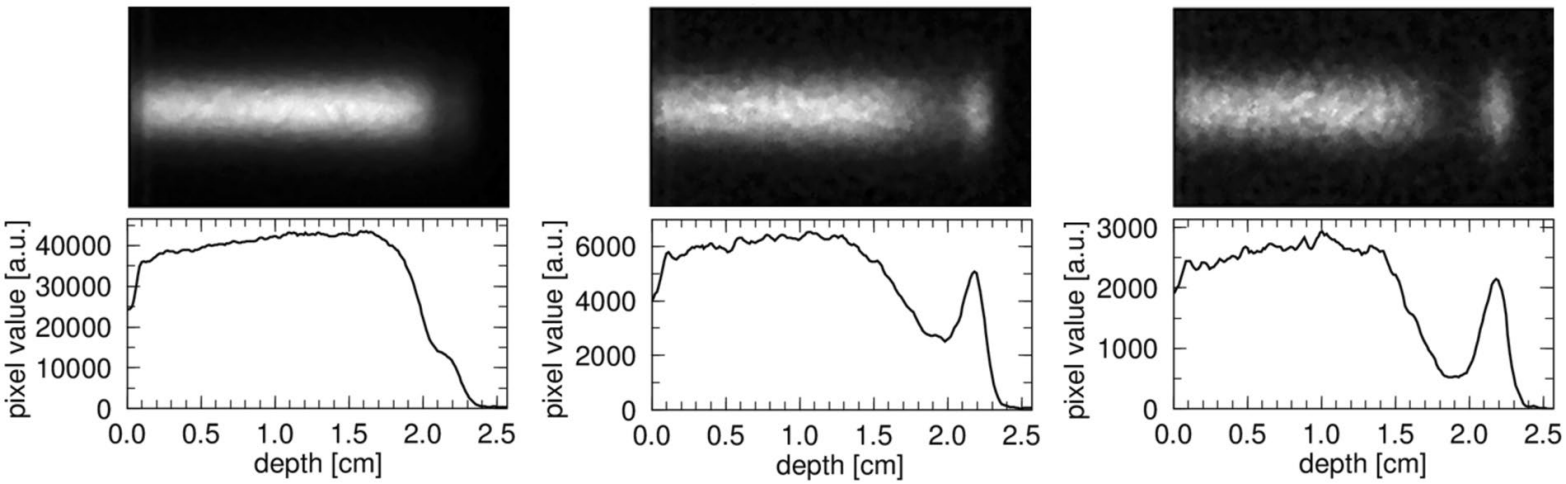
Temporal evolution of the (*upper*) CCD image of the Cherenkov radiation in SiO_2_ and (*bottom*) 1D projection; from left, 120–125 s, 600–605 s, and 1200–1205 s after irradiation.

To measure the proton-induced nuclear reaction cross sections in oxygen, we chose a synthetic quartz glass SiO_2_ as a target. Figure 1 shows the temporal evolution of EM-CCD image, covering 120 s to 1205 s after the proton irradiation. The background subtractions followed by the median filter processing was performed for each image. Clearly, the distribution of Cherenkov radiation gradually changed with time. Note that intensity of the image, represented by “pixel value”, decreased with time. Moreover, the peak emission, which was hardly visible in the left panel, newly appeared in the middle and right panels, suggesting that the dominant positron emitters changed as time passed, due to the different decay times.

**Figure 2:**
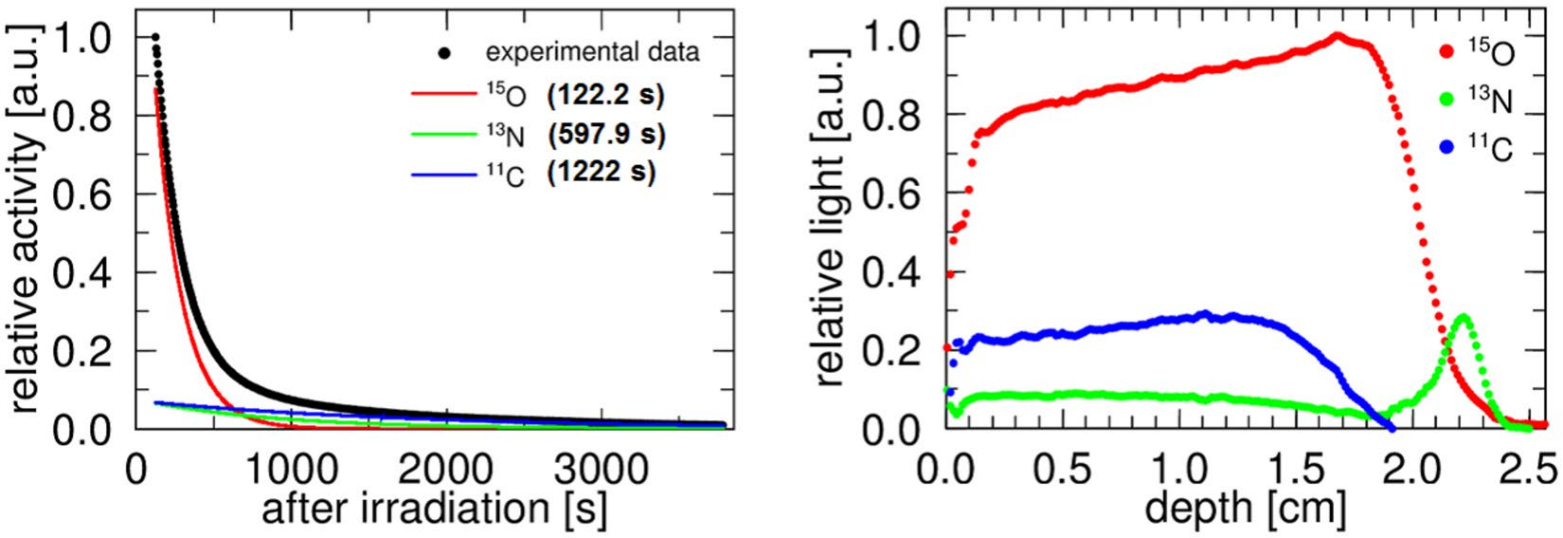
(*left*) Measured decay curve with three fitting curves and (*right*) the resolved distribution of Cherenkov radiation from ^15^O, ^13^N and ^11^C.

We used data sets of 120 to 3600 s for the analysis to remove the very fast components of positron emitters generated from silicon (half-lives: under 10 s). The positron emitters generated from oxygen were ^14^O, ^15^O, ^13^N, and ^11^C, with half-lives 70.62, 122.2, 597.9, and 1222 s^25^, respectively, in the energy range of proton therapy^11,12^. In this study, ^15^O, ^13^N, and ^11^C were considered to contribute to the Cherenkov radiation because the half-life of ^14^O is shorter than 120 s and the cross section values of ^14^O were very small (estimated at a few mbarns). By fitting the decay curve (see Supplementary information) at each depth, we resolved the contribution of each of the three components, namely, ^15^O, ^13^N, and ^11^C. An example of the decay curve fitting is shown in Fig. 2 (*left*) and the obtained distributions of the Cherenkov radiation decomposed by the decay curve fitting are presented in Fig. 2 (*right*). Note that the sudden drop in Cherenkov intensity at a depth below 0.12 cm was artificial, as the irradiated sample had a finite size, and the slightly different viewing angles, when measuring with the EM-CCD, affected the edge region of the target image.

### Nuclear reaction cross sections

**Figure 3:**
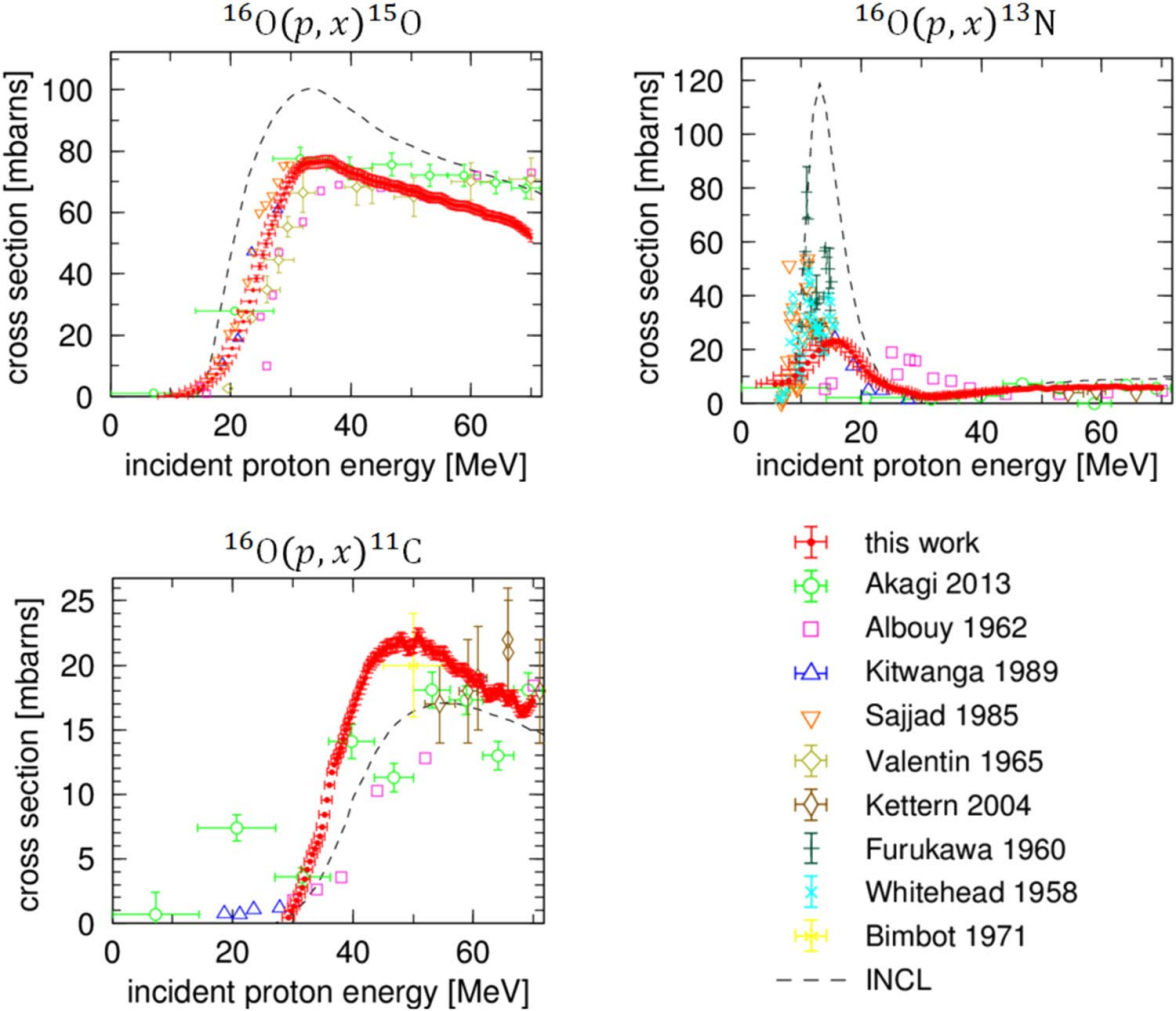
Cross sections of (*upper left*) ^16^O(*p*,*x*)^15^O, (*upper right*) ^16^O(*p*,*x*)^13^N, and (*bottom left*) ^16^O(*p*,*x*)^11^C. The previous experimental values are reported in the NNDC.

By processing the Cherenkov blur deconvolution and flux correction (see Supplementary information), we acquired the relative cross section values of each of the positron emitters. As the normalization of the Cherenkov radiation intensity as measured by the EM-CCD was arbitrary, the resultant cross sections were only given in relative units and difficult to quantify. As the first attempt in this study, we used the archival data of ^16^O(*p,x*)^15^O^23,26^ that was the most densely sampled with the least uncertainty, and the results agreed with others in literature. Note that the acquired cross section and archival data peaked at almost same energy, 35 MeV. Therefore, we normalized the acquired cross sections determined from the Cherenkov radiation to match with the archival maximal cross section of 76.8 mbarns ± 2.45% at 35 MeV. By choosing an absolute value, the cross sections of ^16^O(*p,x*)^15^O, ^16^O(*p,x*)^13^N, and ^16^O(*p,x*)^11^C were determined simultaneously. In this way, we acquired the three nuclear reaction cross sections in the energy range of 5–70 MeV. They are listed in Supplementary Table. We compared the acquired cross sections to those reported in the National Nuclear Data Center (NNDC)^23,26–33^ and the Intra-Nuclear Cascade of Liège (INCL) code^34^ as a general nuclear reaction model (Fig. 3). It is notable that our acquired cross sections have many more data points, smaller deviations, and smaller error values than the previous experimental values, as long as the normalization of the cross section is performed correctly.

We compared our data with previous experimental values and refer to the INCL briefly. Our data for ^16^O(*p*,*x*)^15^O around 10–30 MeV was consistent with previous experimental values^23,26,28,29^ within 1-*σ* uncertainty. In the energy range over 40 MeV, our acquired cross section values gradually decreased. This trend was more significant compared to that observed by Akagi *et al*., Albouy *et al*, and Valentin. It is noted that this tendency was also supported by other research groups^35^ and is also verified in the next subsection. The trend of the INCL was similar to that of our acquired cross section, but the absolute value was approximately 30% larger.

^16^O(*p*,*x*)^13^N is considered to be the sum of ^16^O(*p*,*α*)^13^N and ^16^O(*p*,2*p*2*n*)^13^N. The distinct features seen in the low energy range of approximately 10–20 MeV of ^16^O(*p*,*α*)^13^N were due to a combination of multiple peaks, explained by the nuclear resonance between ^16^O and a proton. Compared to Sajjad *et al*., Furukawa *et al*., and Whitehead *et al*., the peak width was broader and the peak value was approximately three times smaller in our data. This discrepancy can be explained by the energy resolution. There were several excitation energies of ^16^O(*p*,*α*)^13^N around 10–20 MeV. Our acquired cross section values for 10–20 MeV have uncertainties of a few MeV due to the straggling effect, and the typical width of the nuclear resonance line was several hundred keV; therefore, narrow peaks formed by the excitation energies were not separated in our data. As the straggling effect is also observed at the time of the actual proton therapy, our data is applicable, and this is discussed further in the next subsection. For higher energies, greater than 30 MeV, our data was consistent with that of Akagi *et al*., Kitwanga *et al*., Kettern *et al*., and the INCL. A gradual increase from 30 to 50 MeV was considered to be contamination from the ^16^O(*p*,2*p*2*n*)^13^N, with a threshold energy of approximately about 30 MeV.

The acquired cross section values of ^16^O(*p*,*x*)^11^C were roughly consistent with the previous experimental values and the INCL, but had a sharp gradient for the increasing area near 30–45 MeV. Considering the sparse data points and large uncertainties in previous reports, we succeeded in performing the precise measurement of the ^16^O(*p*,*x*)^11^C cross section.

### Comparison with PET measurement

First, we simulated the PET measurement of water using the INCL cross section, which was used in various MC simulation codes^36^. We refer to the current status of the MC simulation for PET-based proton therapy. Then, to verify the newly obtained cross sections, we implemented them as a new library of the PHITS simulation code and compared these to the PET measurements.

**Figure 4:**
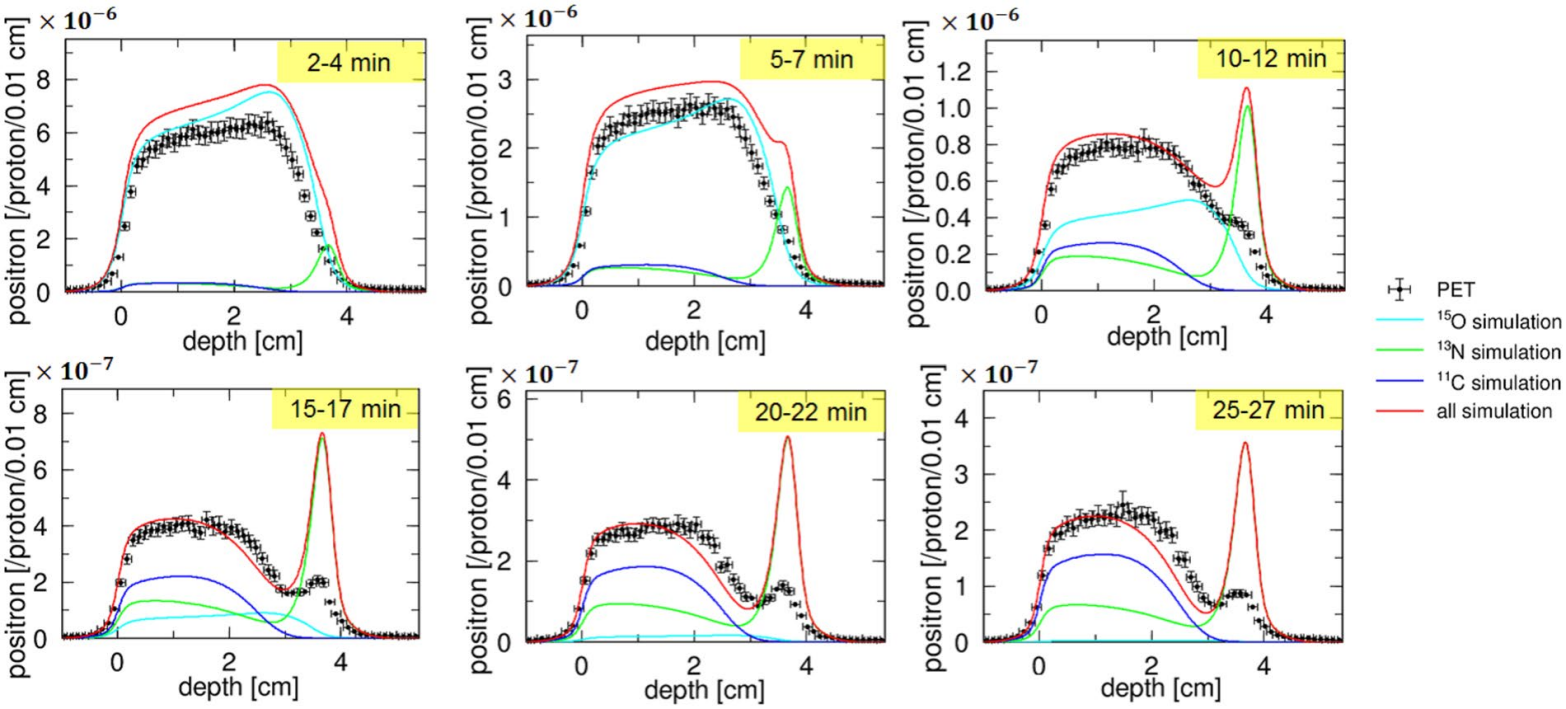
Comparison of the MC simulation, with a nuclear reaction model using the INCL code, with the PET measurements. The graph shows the number of positrons generated by an incident proton; 2–4 min, 5–7 min, 10–12 min, 15–17 min, 20–22 min, and 25–27 min, after irradiation.

**Figure 5:**
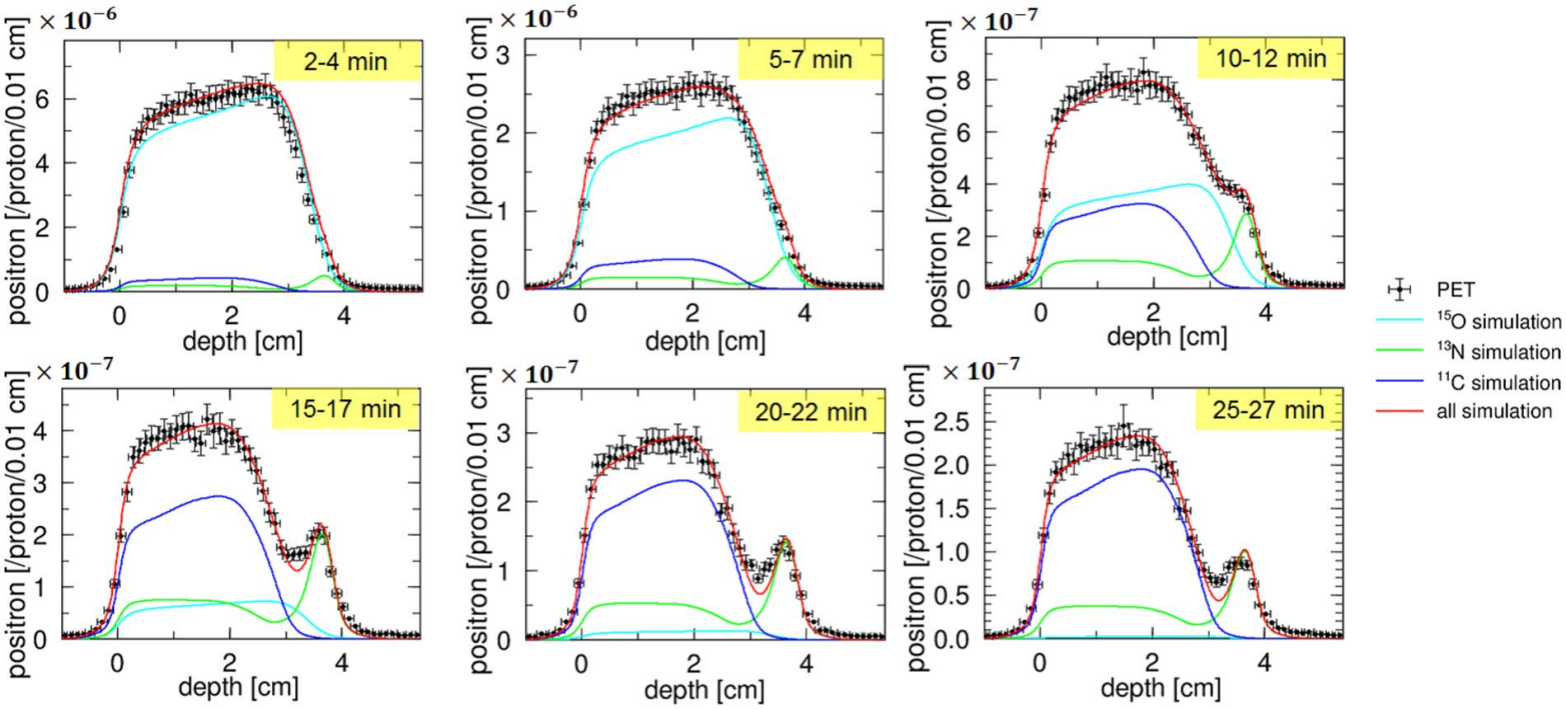
Comparison of MC simulation, implemented using our acquired cross sections, with the PET measurements. The graph shows the number of positrons generated by an incident proton; 2–4 min, 5–7 min, 10–12 min, 15–17 min, 20–22 min, and 25–27 min, after irradiation.

In the PET measurements, the total exposure dose was 95.5 Gy, corresponding to 1.96 × 10^11^ of the incident protons. We then measured the number of positrons generated by an incident proton at each depth. The results are presented in Figs. 4 and 5, and all results took into account the statistic and systematic uncertainties.

It was clear that the existing nuclear reaction model was not valid in the energy range of proton therapy. An accurate estimation of the dose from the PET image was not possible with using these data. In contrast, our MC simulation results at each time interval were in good agreement with the PET measurements, within 1-*σ* uncertainty, for all regions. This result validates the normalization method and the obtained cross section values of ^16^O(*p*,*x*)^15^O, ^16^O(*p*,*x*)^13^N, and ^16^O(*p*,*x*)^11^C. We focused on the peak values at a depth of approximately 3.6–3.7 cm, where ^16^O(*p*,*x*)^13^N was dominant. The time evolutions of the PET measurements and our MC simulation around the peak were consistent, suggesting that our measured ^16^O(*p*,*x*)^13^N data was accurate enough to reproduce the PET imaging data. On the other hand, a small discrepancy at a depth near 3.0–3.2 cm was observed at 15–17 min, 20–22 min, and 25–27 min. It is probable that the sensitivity correction of the PET detector was not performed precisely. We conclude that our acquired cross sections are promising choice for realizing the proton dose estimation from the PET images.

## Methods

### Principles of measuring the cross sections from Cherenkov radiation

Protons that exceed the Coulomb barrier bombard a target nucleus, some of which produce positron emitters via various nuclear reaction channels. Positrons can therefore emit weak light along their path, namely Cherenkov radiation^37,38^, which has been receiving attention as a new tool for luminescence imaging^39,40^. Therefore, the observed light after proton irradiation traces the distribution of generating positron emitters^41–43^ and the temporal evolution of the Cherenkov radiation reflects the decay time. As protons travel through the material while depositing energy, the depth distribution can be converted into the proton energy distribution. Then, by acquiring the light intensity in the depth direction, we can convert it into the nuclear reaction cross section depending on the incident proton energy.

We acquired the spatial and temporal evolution of Cherenkov radiation by using a CCD camera as the detector. We plotted the pixel values versus the time at each depth. By directly fitting a decay curve, we resolved the contributions of each of the positron emitters. The distribution of the resolved Cherenkov radiation was slightly different from the spatial distribution of the positron emitters themselves, because Cherenkov radiation is generated along the tracks of positrons. We then performed Cherenkov blur deconvolution, and converted the depth into the proton energy. It is noted that the total proton flux gradually decreased with increasing depth, which was also corrected in our calculation. The absolute values of the cross sections could not be determined. Hence, we used the archival data of ^16^O(*p*,*x*)^15^O in the NNDC.

In this study, we used the MC simulation code PHITS Ver.2.93, which was mainly developed by the Japan Atomic Energy Agency (JAEA)^44^, and the nuclear reaction model INCL Ver.4.6^34^. The INCL code describes protons, neutrons, pions and light-ions(*d*, *t*, ^3^He, or *α*) induced reactions. This code is also available in the Geant4 particle-transport toolkit and Monte Carlo N-Particle Transport Code (MCNPX)^36^. To calculate all reactions precisely, the event generator mode^45–47^ Ver.2 was also used.

### Experimental setup

We performed two differential experiments, both of which were conducted at the National Institute of Radiological Sciences (NIRS), Japan, and the protons were provided by the AVF930 cyclotron.

**Figure 6:**
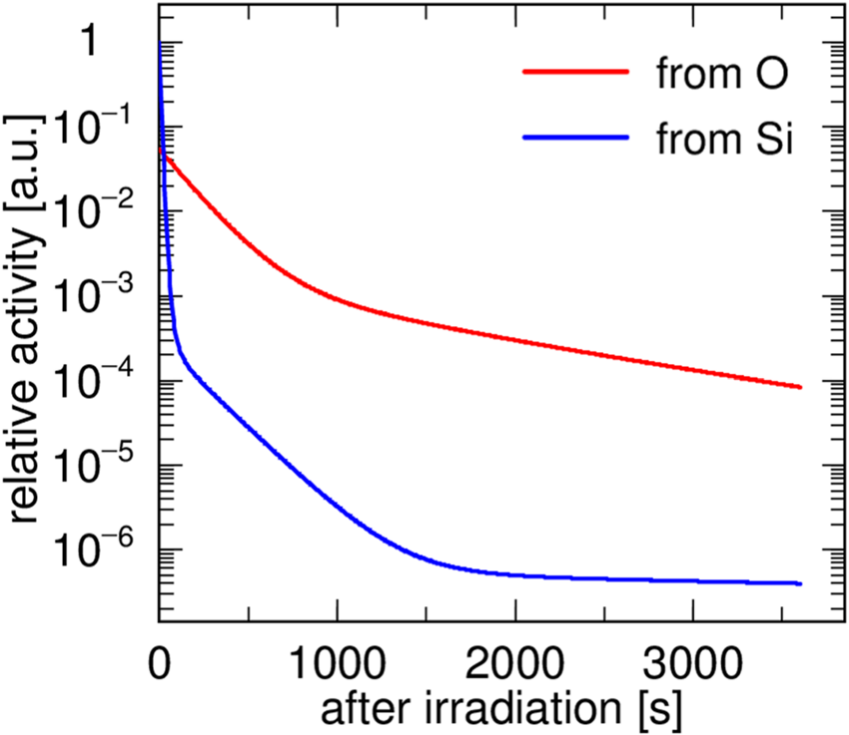
Expected activity of positron emitters from oxygen and silicon in SiO_2_ simulated by PHITS.

To measure the Cherenkov radiation, we used an EM-CCD camera (BU-66EM-UV, BITRAN) with a C-mount F-1.4 lens (LM16JC1MS, Kowa Optical Products). The Cherenkov radiation was so weak that the EM gain was configured at a maximum 20, with a cooling temperature of −20 °C and exposure time of 5 s. In the energy range of proton therapy, the nuclear fragmentation between hydrogen and protons does not occur, so that oxygen is the main component of the positron emitters for the PET monitor. We chose a 3 × 3 × 3.5 cm^3^ synthetic quartz glass SiO_2_ as a target, which contained only a very small amount of impurities, of the order of ppb. The decay time of the positron emitters from the silicon and oxygen were completely different^30^. Therefore, the contamination of the positron emitters from the silicon was less than 1%, at 120 s after irradiation (Fig. 6). SiO_2_ is a transparent material, enabling the Cherenkov radiation to be measured from outside the material by the CCD camera. The distance between the target and the CCD camera was 50 cm, where the pixel length of the CCD camera corresponded to 133 *μ*m. The target was shielded from light except for the readout surface, and the measurement was performed with the experimental room completely dark. The SiO_2_ was irradiated with a 72.0-MeV pencil beam at 30 nA for 30 min. The beam size was 0.400 × 0.246 cm^2^ (FWHM). After the proton irradiation, we measured the Cherenkov radiation for an hour.

**Figure 7:**
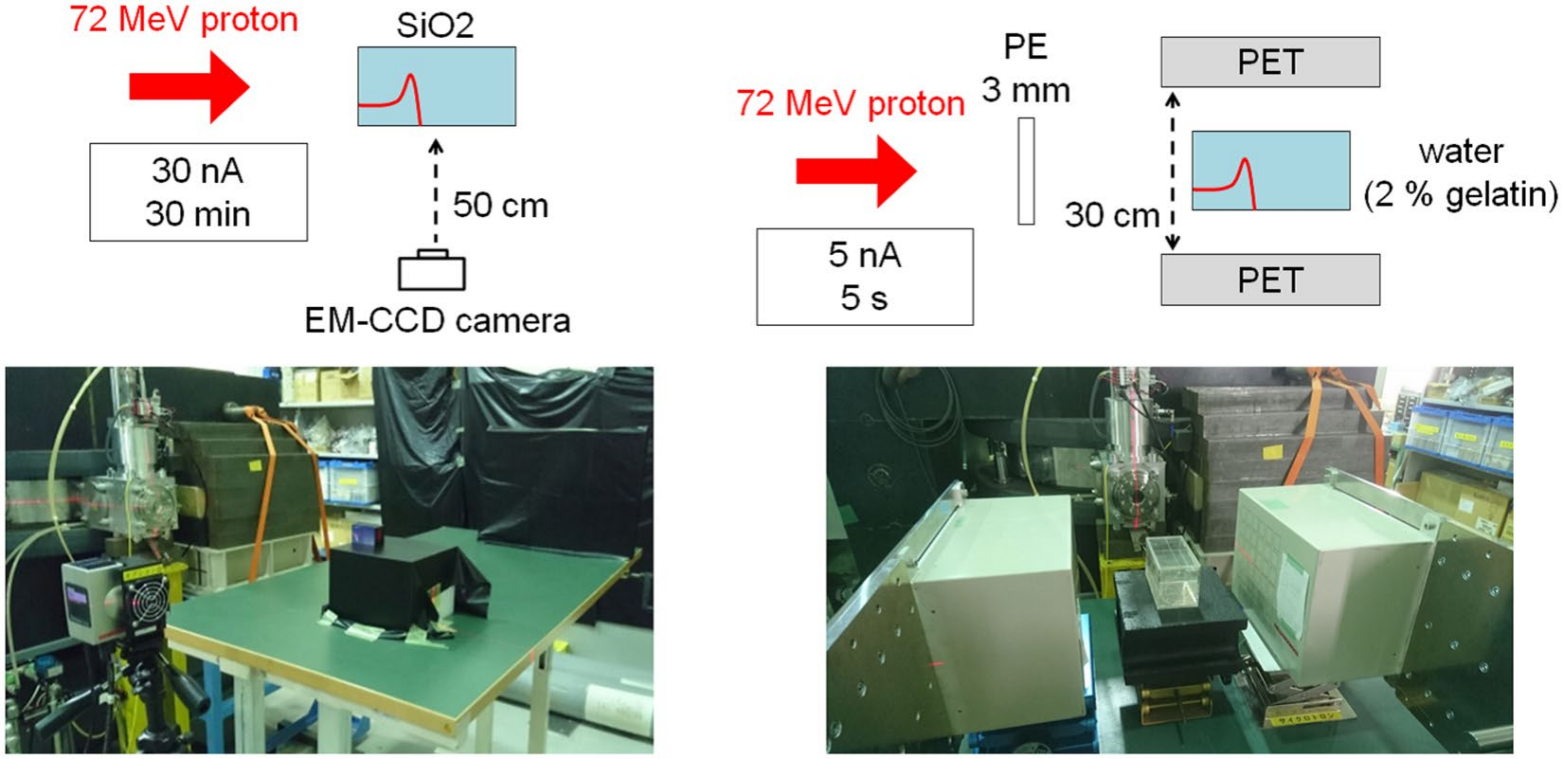
Experimental setup of (*left*) Cherenkov measurement and (*right*) PET measurement.

To verify our acquired cross sections, we also performed a PET measurement of a water target. We used planar-type PET (PPIS-4800-01, Hamamatsu Photonics)^48^, whose field of view (FOV) is 16.72 × 16.5 cm^2^. In each detector unit, 36 blocks of Position Sensitive PMT (R8520-00-C12, Hamamatsu Photonics) were equipped with 11 × 10 BGO scintillators. Each BGO scintillator of size 2.0 × 2.0 × 20 mm^3^ ware placed with a 2.2 mm pitch. The distance between the two detectors was 30 cm with a spatial resolution of 2.0 mm at FWHM. The absolute value of the PET count was calibrated with a ^22^Na point source (96.3 kBq ± 3%). The water target with 2% gelatin was contained by a 6.6 × 6.6 × 12.6 cm^3^ acrylic container with a thickness of 3 mm. To eliminate the influence of the positron emitters from the acrylic, the beam entrance region was covered with only a very thin film. The water was irradiated with a 72.0-MeV pencil beam at 5 nA for 5 s, with the 3 mm polyethylene (PE) inserted on the upstream side to decelerate the incident protons, whose energy decreased to 69.2 MeV. The number of incident protons were evaluated using a beam monitor, which was calibrated with an ionization chamber (Markus Ion Chamber 23343, PTW) in advance. After the proton irradiation, the PET measurement was performed for 30 min. These two experimental setups are illustrated in Fig. 7.

### Data availability

The acquired cross sections of ^16^O(*p*,*x*)^15^O, ^16^O(*p*,*x*)^13^N, and ^16^O(*p*,*x*)^11^C are included in this Supplementary information files. The other datasets generated and analyzed during the current study are available from the corresponding author on reasonable request.

## Electronic supplementary material

Supplementary information

Table S1
