# Supplementary material for: Measurement of nuclear reaction cross sections by using Cherenkov radiation toward high-precision proton therapy
Source: Sci Rep. 2018 Feb 7;8:2570. doi: 10.1038/s41598-018-20906-z (PMC5803244; doi:10.1038/s41598-018-20906-z)
Supplement: Supplementary file 1 — Supplementary information [file 41598_2018_20906_MOESM1_ESM.pdf]

Supplementary information

Measurement of nuclear reaction cross sections by using Cherenkov radiation toward high-precision proton therapy

Takamitsu Masuda[1,2,*], Jun Kataoka[1], Makoto Arimoto[1], Miho Takabe[1], Teiji Nishio[2], Keiichiro Matsushita[3], Tasuku Miyake[4], Seiichi Yamamoto[5], Taku Inaniwa[6], and Toshiyuki Toshito[7]

[1]Waseda University, Graduate School of Advanced Science and Engineering, Tokyo, Japan
[2]Tokyo Women's Medical University, Department of Medical Physics, Tokyo, Japan
[3]Kyoto Prefectural University of medicine, Department of Radiology, Kyoto, Japan
[4]Rikkyo University, Graduate School of Science, Tokyo, Japan
[5]Nagoya University, Graduate School of Medicine, Nagoya, Japan
[6]National Institute of Radiological Sciences, QST, Department of Accelerator and Medical Physics, Chiba, Japan
[7]Nagoya Proton Therapy Center, Nagoya, Japan
[*]www.my-address@akane.waseda.jp

Equation of decay curve fitting

First, we consider a positron emitter i. The activity of the positron emitter just after the irradiation is stopped, denoted as $A_i(0)$, is described as

$$A_i(0) = \sigma_i \phi N_t \left(1 - \mathrm{e}^{\frac{-\ln 2}{\tau_i} T}\right) \tag{1}$$

where σ_i is the production cross section of the positron emitter i, ϕ is the proton flux, N_t is the number of the nuclide in the target, τ_i is the half-life of the positron emitter i, and T is the irradiation time. After the proton irradiation, the activity of the positron emitter $A_i(t)$ decreases, which is represented by

$$A_i(t) = A_i(0)\mathrm{e}^{\frac{-\ln 2}{\tau_i} t} \tag{2}$$

and the decay number of the positron emitter for t_s to $t_s + t_e$ is

$$A_i(t_s) = \int_{t_s}^{t_s+t_e} A_i(t)dt \tag{3}$$

where t_s is the start time of each measurement and t_e is the exposure time of the CCD camera. Observed Cherenkov radiation from CCD camera $F(t_s)$ is sum of the several kinds of positron emitters. The acquired intensity is represented as

$$F(t_s) \propto \sum_i C_i A_i(t_s) \tag{4}$$

where C_i is the Cherenkov radiation weighting factor as detailed below. This factor corrects the differential number of photons emitted as Cherenkov radiation due to the differential energy spectrum of the positrons. Thus, combined with equation 1, 2, 3, and 4, $F(t_s)$ is

$$F(t_s) \propto \sum_i C_i \sigma_i \phi N_t \frac{\tau_i}{\ln 2} f \left(1 - \mathrm{e}^{\frac{-\ln 2}{\tau_i} T}\right) \left(\mathrm{e}^{\frac{-\ln 2}{\tau_i} t_s} - \mathrm{e}^{\frac{-\ln 2}{\tau_i}(t_s+t_e)}\right) \tag{5}$$

The only unknown quantity is σ_i in the equation 5. By performing decay curve fitting at each depth, we can determine the relative values of the cross sections that produce positron emitters corresponding to the proton energy. In this paper, the standard errors of $\sigma_{^{15}\mathrm{O}}$, $\sigma_{^{13}\mathrm{N}}$, and $\sigma_{^{11}\mathrm{C}}$ were 0.340-2.80 %, 0.479-3.93 %, and 0.556-1.28 % respectively.

Cherenkov radiation weighting factor C_i

In this section, we evaluate the number of photons emitted as Cherenkov radiation by each positron emitter, defined as the Cherenkov radiation weighting factor, C_i. First, we calculate the number of Cherenkov photons from a positron having initial energy E_{in}. Next, we consider the theoretical energy spectrum of the positron emitters. By combining these two factors, we obtain the value of C_i.

In the relativistic sense, a requirement of generating Cherenkov radiation is defined as

$$E \geqq mc^2 \left(\frac{1}{\sqrt{1-\frac{1}{n^2}}} - 1 \right) \tag{6}$$

where n is the refractive index of the medium. From the positron, the Cherenkov radiation is emitted at a particular angle θ defined as

$$\cos\theta = \frac{1}{\beta n} \tag{7}$$

where β is the velocity of the positron, defined as

$$\beta = \sqrt{1 - \left(\frac{mc^2}{E + mc^2} \right)^2} \tag{8}$$

in the relativistic sense. The number of emitted photons N depends on the wavelength band from λ_1 to λ_2 and a particular angle θ. It is described per unit path length dx as

$$\frac{dN}{dx} = 2\pi\alpha \sin^2\theta \int_{\lambda_1}^{\lambda_2} \frac{1}{\lambda^2} d\lambda \tag{9}$$

and equation 9 is integrated as

$$\frac{dN}{dx} = 2\pi\alpha \sin^2\theta \left(\frac{1}{\lambda_1} - \frac{1}{\lambda_2} \right) \propto \sin^2\theta \tag{10}$$

Equation10 can be interpreted as follows: the number of photons emitted per unit path length dN/dx depends only on a particular angle θ. Therefore, by combining Equations 7, 8, and 10, the number of photons emitted per unit path length dN/dx depends only on the kinetic energy of the positron E, described as

$$\frac{dN}{dx} = 2\pi\alpha \left(\frac{1}{\lambda_1} - \frac{1}{\lambda_2} \right) \left(1 - \frac{1}{n^2} \frac{1}{1 - \left(\frac{mc^2}{E+mc^2} \right)^2} \right) \tag{11}$$

Using Equation 11 and the positron energy-range Table[1], the number of Cherenkov photons emitted by a positron at initial energy E_{in} is calculated (Fig. S1 $(left)$).

The theoretical energy spectrum of the positron generated via β^+ decay, $N(E)$, is defined as

$$N(E) = g(E + mc^2)\sqrt{E^2 + 2mc^2 E}(E_{max} - E)^2 F(Z, E) \tag{12}$$

where g is a coupling constant determining the power of particle interaction, E is the kinetic energy of the positron, E_{max} is the maximum kinetic energy of the positron, mc^2 is the rest energy of the positron, and Z is the atomic number of the daughter nucleus[2–4]. $F(Z,E)$ is the Fermi function defined as

$$F(Z,E) = \frac{2\pi\eta}{1 - \mathrm{e}^{-2\pi\eta}} \tag{13}$$

with

$$\eta = -\frac{Z\alpha(E + mc^2)}{\sqrt{(E^2 + 2mc^2 E)}} \tag{14}$$

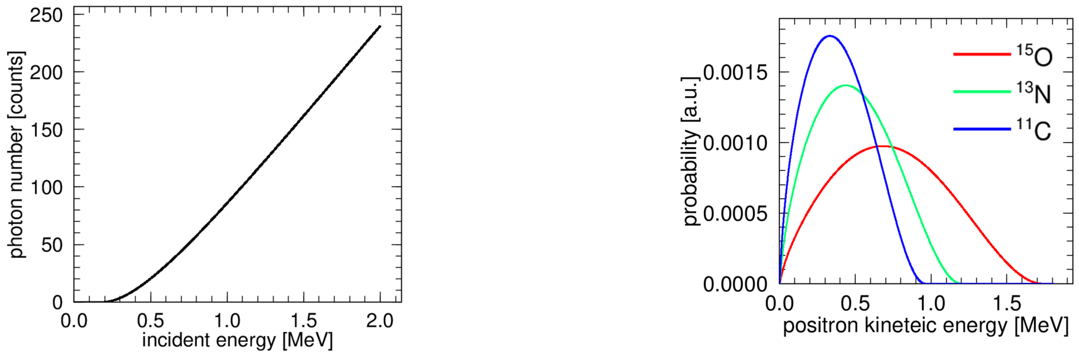

Figure S1. $(left)$ Number of Cherenkov photons emitted by a positron at initial energy E_{in} and $(right)$ the energy spectrum of the positron emitted by ^{15}O, ^{13}N, and ^{11}C

where α is the fine-structure constant. The Fermi function represents the Coulomb interaction between the positron and daughter nucleus. According to Equations 12, 13, and 14, the theoretical energy spectrum of the positron emitted by ^{15}O, ^{13}N, and ^{11}C is shown in Fig. S1 $(right)$.

By combining the number of Cherenkov photons emitted by a positron (Fig. S1 $(left)$) and the energy spectrum of the positron (Fig. S1 $(right)$), we acquire the Cherenkov radiation weighting factor C_i. The refractive index n and the density ρ of the synthetic quartz glass SiO_2 are 1.47 and 2.21, respectively; thus, the calculated values for $C_{^{15}\mathrm{O}}$, $C_{^{13}\mathrm{N}}$, and $C_{^{11}\mathrm{C}}$ are 93.97, 43.16, and 24.87, respectively.

Conversion of depth profiles of Cherenkov radiation into cross sections

The distribution of the resolved Cherenkov radiation is not equivalent to the production distribution of the positron emitters because the Cherenkov radiation is generated along the tracks of the positrons. Therefore in this study, we defined the point spread functions (PSF) as the expanse of Cherenkov radiation from each positron emitter (Fig. S2 $(left)$) and performed an Wiener deconvolution with a regularization parameter of 0.1. The calculated proton flux is shown in Fig. S2 (*center* and *right*). The center plot represents the change in proton flux with respect to the depth, enabling the correction of the number of incident protons at each depth. The right plot describes the energy distribution of protons at a depth of 2.00 cm, which is asymmetrically broad owing to energy straggling. The proton energy at each depth was converted and the argument of the maximum was defined as the incident proton energy E. The uncertainties δE_{Lower} and δE_{Higher} were determined by the $\pm$ 34 % area at the lower and higher energy sides, corresponding to 1-σ values of the Gaussian function. The flux uncertainty gradually increased with depth.

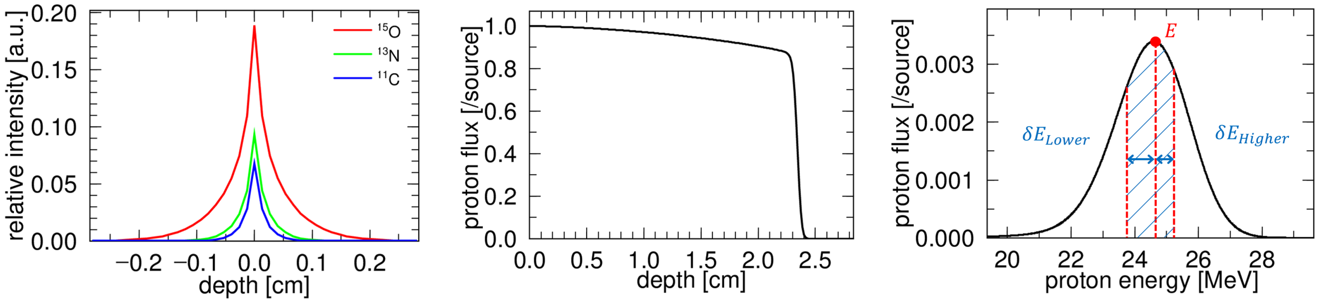

Figure S2. Correction factors for converting Cherenkov radiation into cross sections. $(left)$ The estimated PSF of the Cherenkov radiation, $(center)$ change in proton flux with respect to the depth, and $(right)$ energy distribution of protons at depth 2 cm. In the *right* panel, the striped area corresponds to the 1-σ area of the Gaussian function.

References

1. Institute of Standards, N. & Technology (NIST). Stopping-Power & Range Tables for Electrons, Protons, and Helium Ions. `https://www.nist.gov/pml/stopping-power-range-tables-electrons-protons-and-helium-ions`.

2. Wu, C. S. & Moskowski, S. A. *Beta Decay* (Interscience Publishers, New York, 1966).

3. Daniel, H. Shapes of beta-ray spectra. *Rev. Mod. Phys.* **40**, 659–672 (1968).

4. Levin, C. S. & Hoffman, E. J. Calculation of positron range and its effect on the fundamental limit of positron emission tomography system spatial resolution. *Phys. Med. Biol.* **44**, 781–799 (1999).
